# Neuroantigen-specific, tolerogenic vaccines: GM-CSF is a fusion partner that facilitates tolerance rather than immunity to dominant self-epitopes of myelin in murine models of experimental autoimmune encephalomyelitis (EAE)

**DOI:** 10.1186/1471-2172-12-72

**Published:** 2011-12-30

**Authors:** Derek J Abbott, J Lori Blanchfield, David A Martinson, Sean C Russell, Najla Taslim, Alan D Curtis, Mark D Mannie

**Affiliations:** 1 The Department of Microbiology and Immunology, East Carolina University, Greenville, NC, USA; 2 The Department of Microbiology and Immunology, Emory University, Atlanta, GA, USA

## Abstract

**Background:**

Vaccination strategies that elicit antigen-specific tolerance are needed as therapies for autoimmune disease. This study focused on whether cytokine-neuroantigen (NAg) fusion proteins could inhibit disease in chronic murine models of experimental autoimmune encephalomyelitis (EAE) and thus serve as potential therapeutic modalities for multiple sclerosis.

**Results:**

A fusion protein comprised of murine GM-CSF as the N-terminal domain and the encephalitogenic MOG35-55 peptide as the C-terminal domain was tested as a tolerogenic, therapeutic vaccine (TTV) in the C57BL/6 model of EAE. Administration of GMCSF-MOG before active induction of EAE, or alternatively, at the onset of EAE blocked the development and progression of EAE. Covalent linkage of the GM-CSF and MOG35-55 domains was required for tolerogenic activity. Likewise, a TTV comprised of GM-CSF and PLP139-151 was a tolerogen in the SJL model of EAE.

**Conclusion:**

These data indicated that fusion proteins containing GM-CSF coupled to myelin auto-antigens elicit tolerance rather than immunity.

## Background

In most patients, multiple sclerosis (MS) initially presents as a relapsing-remitting disease course that is marked by periodic, self-limiting attacks interspersed among prolonged periods of apparent clinical latency [1-5]. Although the etiology of MS is not understood, a prevalent theory is that molecular mimicry drives the encephalitogenic attack [6-8]. Molecular mimicry may be mediated by chronic infectious agents such as viruses that exhibit prolonged latency but periodically reactivate and consequently re-stimulate cross-reactive immunity. With each reactivation, these chronic infectious agents may elicit a new wave of effector and memory T cells with cross-reactive specificity for viral epitopes and self epitopes of CNS myelin. Focal infiltration of cross-reactive T cells into the CNS is coupled with T cell-reactivation upon recognition of the cross-reactive self-myelin antigens [9,10]. This process in turn drives inflammatory demyelination and neurologic dysfunction. These inflammatory processes are then postulated to elicit negative feedback pathways and compensatory regulatory responses that enable spontaneous remission and recovery. In many patients, this relapsing-remitting form of MS evolves into a chronic progressive disease in which periodic attacks are subsumed by an insidious and continuous deterioration of neurological function [11-14]. This transition from an inflammatory relapsing-remitting disease to a progressive neurodegenerative disease is postulated to reflect mechanisms of epitope spreading and erosion of regulatory T cell control. This transition is also marked by a progressive loss in therapeutic efficacy of anti-inflammatory drugs.

EAE is a widely studied animal model of MS [5]. Some models of EAE are characterized by an acute monophasic attack followed by a spontaneous remission and permanent recovery whereas other EAE models exhibit continual relapsing-remitting or chronic progressive courses of disease. Monophasic, self-limiting models of EAE that feature spontaneous, enduring recovery may have more robust regulatory T cell responses compared to those operative in chronic models. Likewise, strategies of antigen-specific tolerance induction may be more successful in monophasic models due to the potential presence of more robust regulatory responses compared to chronic models of EAE.

Cytokine- NAg fusion proteins have been studied in the acute monophasic model of EAE in Lewis rats as potent NAg-specific tolerogens [15-18]. Cytokine-NAg fusion proteins were comprised of IL-2, IL-16, IFN-beta, or GM-CSF as the cytokine domain and the dominant encephalitogenic epitope of myelin basic protein as the NAg domain. When administered before encephalitogenic challenge, these TTV effectively prevented the subsequent induction of EAE. When administered during the onset of clinical signs, the same TTV inhibited disease progression and accelerated remission. Of these TTV, GMCSF-NAg was the most efficient for targeting NAg to rat myeloid APC [15]. An important question is whether TTV-based strategies of tolerance induction are effective in both monophasic and chronic models of EAE, particularly across both rat and mouse species.

In this study, a fusion protein comprised of murine GM-CSF as the N-terminal domain and the encephalitogenic MOG35-55 peptide as the C-terminal domain was tested as a TTV in the C57BL/6 model of EAE. Subcutaneous administration of GMCSF-MOG in saline on days -21, -14, and -7 inhibited the subsequent induction of active EAE. A parallel GMCSF-PLP(139-151) fusion protein was tolerogenic in the SJL model of EAE. Several additional experiments focused on the GMCSF-MOG TTV. When administration was initiated at the onset of clinical signs in actively-immunized mice, GMCSF-MOG prevented the progression of EAE. Covalent linkage of the cytokine and MOG35-55 domains was required for tolerogenic activity. When administered during the course of passively-induced EAE, GMCSF-MOG accelerated recovery and blunted a subsequent active induction of EAE. In conclusion, GMCSF-NAg TTV ameliorated disease in two chronic models of murine EAE. These data support the overall concept that GMCSF-NAg fusion proteins are potent tolerogens in both rat and mouse species and are effective in both monophasic and chronic models of EAE.

## Results

### In vitro activities of GMCSF-MOG

**Figure 1:**
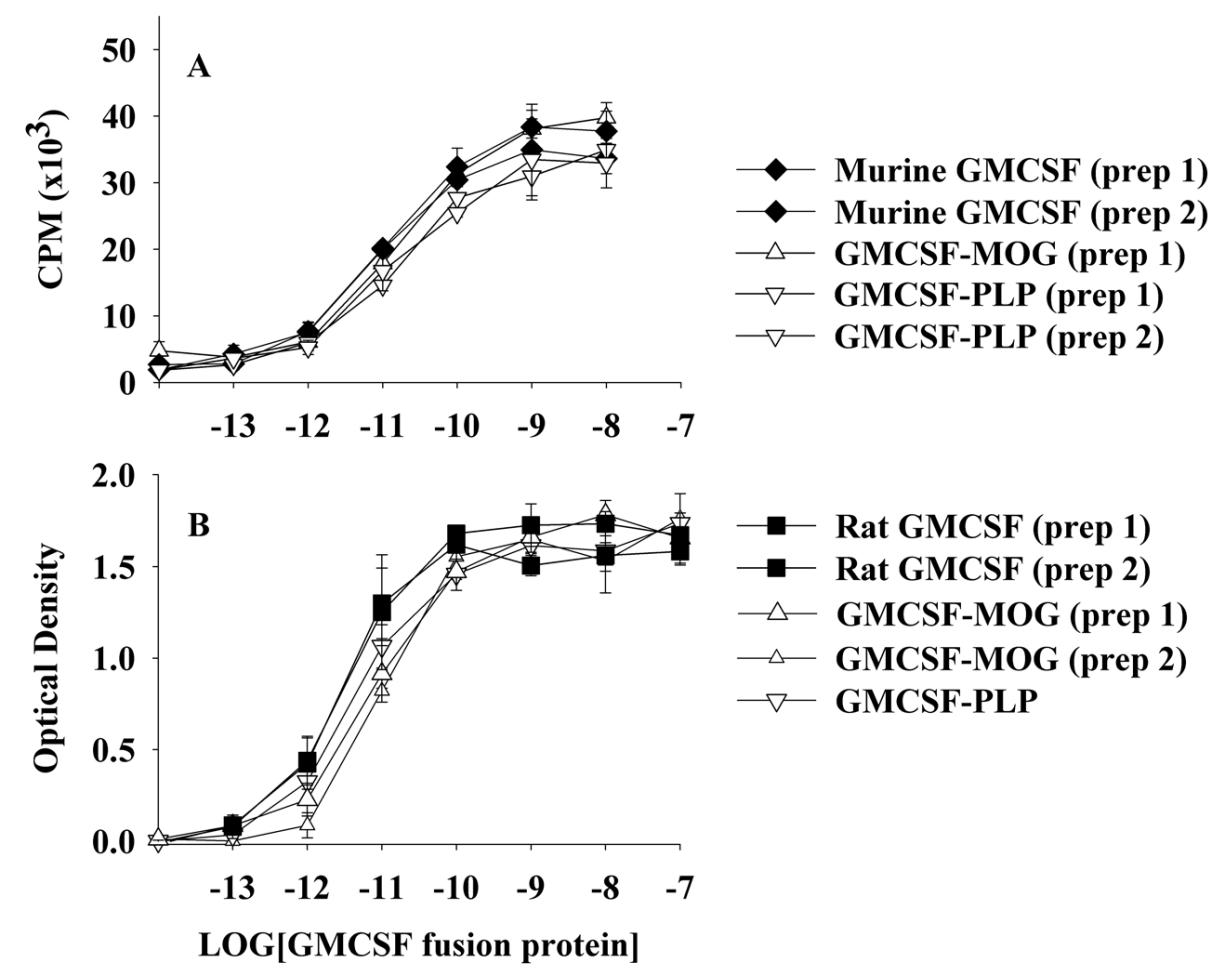
**The NAg domain of murine GMCSF-NAg TTV did not interfere with cytokine bioactivity**. Designated concentrations (x-axis) of murine GM-CSF, rat GM-CSF, murine GMCSF-MOG, or murine GMCSF-PLP were cultured with C57BL/6 bone marrow cells (100,000 cells per well) (A) or FDC-P1 cells (10,000 cells per well) (B). Cultures were pulsed with 1 uCi of [^3^H]thymidine (A) or MTS/PMS (B) during the last 24 hours of a 3-day culture. These data are representative of four experiments.

The main question addressed in this study was whether murine GMCSF-NAg TTV could block disease in murine models of chronic EAE, including the chronic progressive model of EAE in C57BL/6 mice and the relapsing-remitting model of EAE in SJL mice. Murine fusion proteins were derived which consisted of the murine GM-CSF as the N-terminal domain and either the MOG35-55 epitope or the PLP139-151 epitope as the C-terminal domain. We first tested whether the bioactivity of GM-CSF was altered by the C-terminal addition of the NAg domain. The bioassays were based on the use of C57BL/6 bone marrow cells (Figure 1A) and the FDC-P1 cell line (Figure 1B). These assays revealed that the GM-CSF activity of GMCSF-MOG and GMCSF-PLP was essentially equipotent with murine GM-CSF and rat GM-CSF. Independently-derived preparations of each fusion protein were tested to verify reliability of the fusion protein preparations. Sample to sample variation of protein preparations was minimal. The conclusion was that neither the MOG35-55 nor an alternative PLP139-151 C-terminus interfered with the activity of the GM-CSF domain. These and other experiments revealed that mouse and rat GM-CSF proteins were equally cross-reactive on both mouse and rat indicator cells (data not shown).

**Figure 2:**
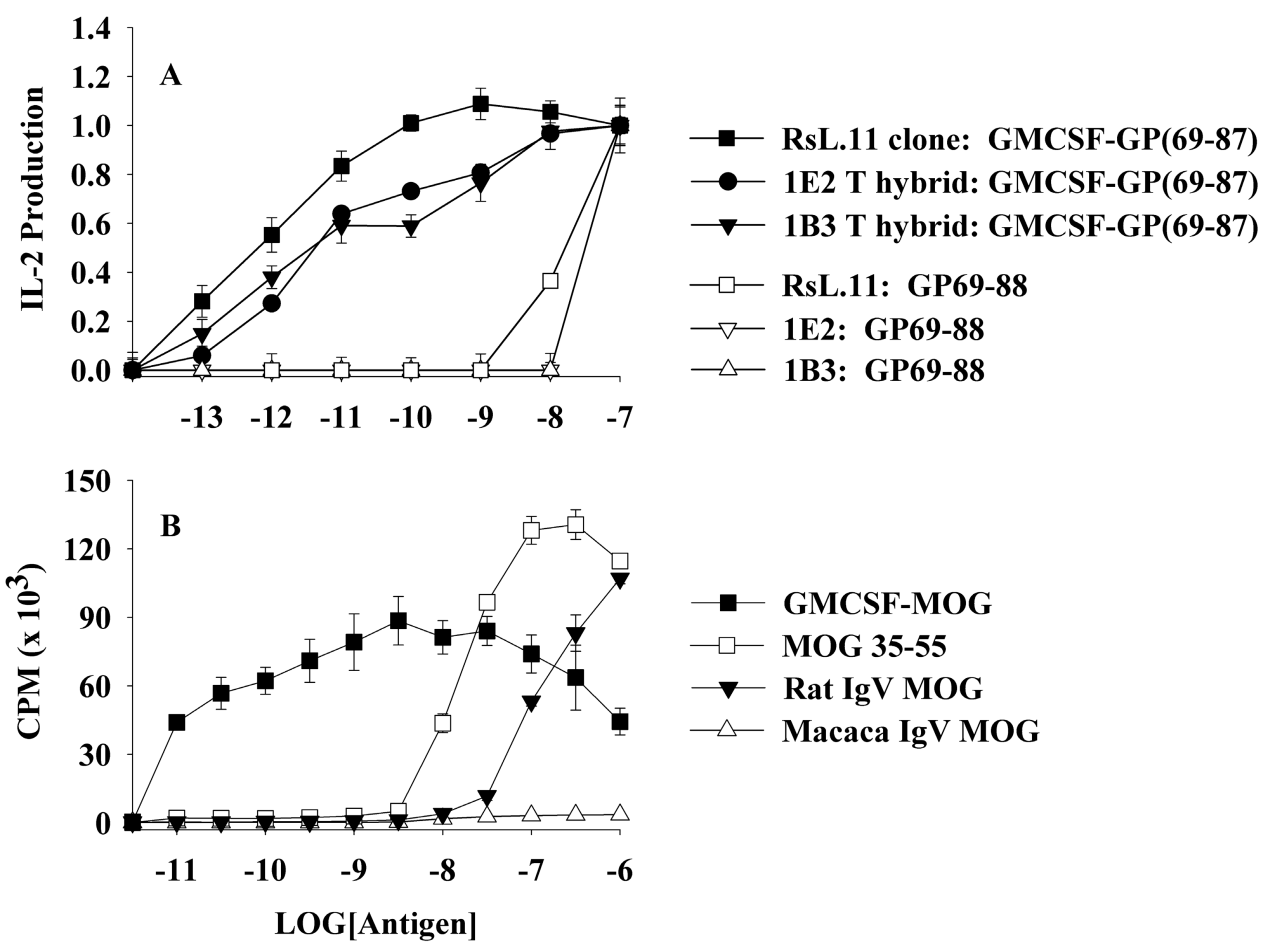
**The GM-CSF domain of murine GMCSF-MOG potentiated the antigenic recognition of MOG35-55 by rat T cells**. (A) The rat T cell clone RsL.11 or the rat 1E2 or 1B3 T cell hybrids were cultured with Lewis rat splenic APC and designated concentrations (x-axis) of the rat GMCSF-(GP69-88) fusion protein or the GP69-88 synthetic peptide. Supernatants were collected after 24 hrs of culture and assayed for IL-2 production. (B) A rat MOG(35-55)-specific T cell line was cultured with rat splenic APC and designated concentrations of murine GMCSF-MOG, the synthetic MOG35-55 peptide, or the IgV extracellular domain of rat or macaca MOG. Cultures were pulsed with 1 uCi of [^3^H]thymidine during the last 24 hours of a 3-day culture. These data are representative of four experiments.

Previous studies also showed that rat GMCSF-NAg TTV were potent antigens in vitro [15]. The enhanced antigenic potency of GMCSF-NAg TTV was due to high affinity interactions of the cytokine domain with the respective cytokine receptors on APC which targeted the tethered NAg to the APC surface for enhanced presentation of NAg. As shown in Figure 2A, the rat GMCSF-NAg TTV was approximately 1000-fold more potent than the guinea pig (GP) myelin basic protein (MBP) GP69-88 peptide when cultured with splenic APC and the RsL.11 T cell clone or the rat 1B3 or 1E2 T cell hybrids. The murine GMCSF-MOG protein also potently enhanced the recognition of the covalently tethered MOG35-55 peptide when assayed in the presence of rat splenic APC and a rat T cell line specific for MOG35-55 (Figure 2B). GMCSF-MOG was at least 1000-fold more potent as an antigen compared to MOG35-55 and 10,000 fold more potent than the extracellular IgV domain of rat MOG which contains the verbatim MOG35-55 sequence. At concentrations of 10 pM (10^-11 ^M) to 1 uM (10^-6 ^M), the stimulatory activity of GMCSF-MOG reflected a broad bell-shaped curve that varied from 43 to 88 (x 10^3^) cpm whereas MOG35-55 was substantially less potent but at high concentrations (100 nM) stimulated a more robust T cell response (130 × 10^3 ^cpm). A recombinant macaca IgV-MOG protein which contained two differences (rat/macaca S42P and K55R) did not stimulate this T cell line. GM-CSF alone did not stimulate proliferative activity (data not shown).

### GMCSF-MOG pre-treatment prevented a subsequent phase of EAE

**Figure 3:**
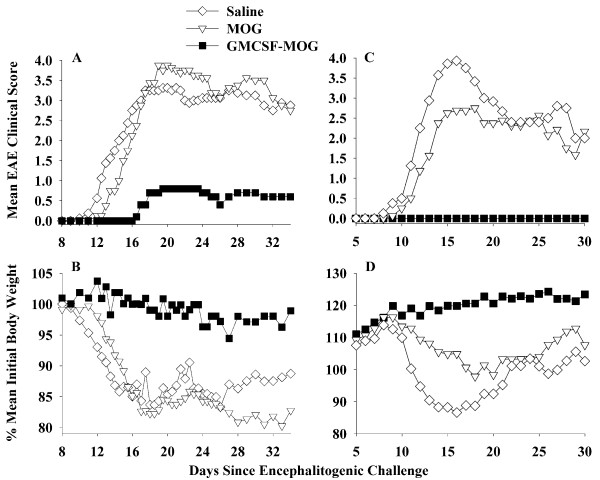
**The murine GMCSF-MOG TTV induced tolerance and prevented a subsequent episode of EAE**. C57BL/6 mice were treated with 2 nmoles of GMCSF-MOG, 2 nmoles of MOG35-55, or saline on days -21, -14, and -7 before active challenge on day 0 with 200 ug of MOG35-55 in CFA and two i.p. injections of Pertussis toxin on days 0 and 2. Table 1 and Figure 3 portray the same experiment. In experiment #1 (A-B), daily clinical disease scores for mice treated with GMCSF-MOG were significantly different from those treated with MOG35-55 (days 16-34). Comparison of "GMCSF-MOG versus saline" groups also revealed significant differences from days 15-19 and 24-28. In experiment #2 (C-D), daily clinical scores for GMCSF-MOG treated mice were significantly different from mice treated with MOG35-55 (days 13-30) or saline (days 11-30).

To assess whether GMCSF-MOG could prevent EAE, the TTV was administered as a pre-treatment regimen (2 nmole dose subcutaneously in saline) at 3, 2, and 1 weeks before encephalitogenic challenge (Table 1 and Figure 3). In experiments 1 and 2 combined, the TTV pretreatment prevented EAE in 12 of 13 mice for an incidence of 7.7%. That is, twelve mice had a maximal score of zero (no disease) and one mouse exhibited severe EAE (maximal score of 4.0). In contrast, mice pretreated with MOG35-55 or saline exhibited an incidence of 100% and 93.8% respectively (Table 1). In the MOG-35-55 pretreated groups, 14 of 16 exhibited severe EAE (≥ 4.0 maximal score) whereas 2 mice exhibited mild EAE (scores of 1.0). In the saline-pretreated groups, 15 of 16 mice had scores of 4.0 whereas one mouse did not exhibit EAE. Thus, the GMCSF-MOG pretreatment reduced the mean cumulative score, mean maximal score, and the mean number of days with severe EAE. The GMCSF-MOG prevented the development of EAE in a majority of mice over a prolonged period of 40-50 days (Figure 3A, C). GMCSF-MOG also prevented the weight loss associated with EAE whereas mice pretreated with MOG35-55 or saline had a sustained loss of body weight (Figure 3B, D). These data indicate that GMCSF-MOG is an active tolerogen in the C57BL/6 mouse model of EAE.

**Table 1:** GMCSF-MOG prevented a subsequent bout of EAE induced by challenge with MOG35-55 in CFA

Exp. #	Pre-treatment^a^	Mean cum. score^b^	Median cum. score^b^	Mean max. score^b^	Median max. score^b^	% mean initial weight^b^	Incidence of EAE^c^	Mean # days with severe EAE^c^
1	Saline	102.6 ± 48.9	115.5	3.5 ± 1.4	4.0	77.9%	7 of 8	18.3 ± 7.6

	MOG	103.8 ± 16.6	106.3	4.0 ± 0.0	4.0	72.5%	8 of 8	18.3 ± 1.8

	GMCSF-MOG	18.3 ± 40.9	0.0	0.8 ± 1.8	0.0	93.6%	1 of 5	3.6 ± 8.0

	TTV vs saline	p = 0.05		p = 0.003		p = 0.015	p = 0.032	p = 0.002

	TTV vs MOG	p = 0.02		p = 0.001		p = 0.001	p = 0.007	p = 0.002

								

2	Saline	41.2 ± 19.4	44.8	4.0 ± 0.0	4.0	84.2%	8 of 8	10.1 ± 5.8

	MOG	40.6 ± 24.3	45.8	3.4 ± 1.5	4.0	92.7%	8 of 8	8.9 ± 6.7

	GMCSF-MOG	0.0 ± 0.0	0.0	0.0 ± 0.0	0.0	103.0%	0 of 8	0.0 ± 0.0

	TTV vs saline	p < 0.001		p < 0.001		p < 0.001	p < 0.001	p = 0.002

	TTV vs MOG	p < 0.001		p < 0.001		p = 0.021	p < 0.001	p = 0.007

^a ^C57BL/6 mice were treated with 2 nmoles of GMCSF-MOG, 2 nmoles of MOG35-55, or saline. Injections were subcutaneous in saline and were given on days -21, -14, and -7 (total combined dose of 6 nmoles) before active challenge on day 0 (200 ug of MOG35-55 in CFA with i.p. injections of Pertussis toxin on days 0 and 2). Table 1 and Figure 3 represent the same experiments. Experiments 1 and 2 correspond to the data shown in Figure 3A-B and 3C-D, respectively.

^b ^Cumulative scores were calculated by summing daily scores for each mouse. Maximal scores were calculated as the most severe EAE score for each mouse. Percent initial body weight was calculated as the minimum weight recorded between day 7 and the end of the experiment divided by the maximum weight recorded from day 0 through day 7. For all tables, the mean cumulative and mean maximal scores included all mice within a group, even those not afflicted by EAE. That is, the score of zero representing mice that did not exhibit EAE was included in the calculation of the respective mean values. Differences in median values for cumulative and maximal scores were analyzed by nonparametric ANOVA based on ranked scores. Differences in mean values for percent initial body weight and "number of days with severe EAE" were assessed by parametric ANOVA. ANOVA was interpreted with the Bonferroni post hoc test. Incidence of EAE was analyzed pair-wise by Fisher's Exact Test.

^c ^Severe EAE was defined as hindlimb paresis or paralysis (clinical score of 3.0 or greater).

**Figure 4:**
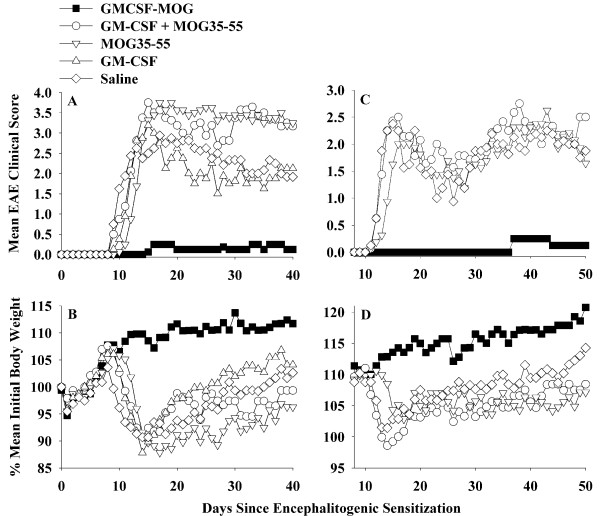
**Induction of MOG-specific tolerance required covalent linkage of the GM-CSF and MOG domains**. C57BL/6 mice were treated with GMCSF-MOG, a mixture of murine GM-CSF and MOG35-55, the MOG35-55 peptide, GM-CSF, or saline subcutaneously on days -21, -14, and -7 (n = 8, all groups, 2 nanomoles per dose). Mice were then immunized on day 0 with 200 ug MOG35-55 in CFA plus Pertussis toxin (200 ng i.p.) on days 0 and 2. Table 2 and Figure 4 represent the same experiments. In experiment #1 (A-B), daily clinical scores for mice treated with GMCSF-MOG were significantly different from those treated with "GM-CSF + MOG35-55" (days 12-42), MOG35-55 (days 14-42), GM-CSF (days 12-42), and saline (days 10-52). In experiment 2 (C-D), daily clinical scores for mice treated with GMCSF-MOG were significantly different from those treated with "GM-CSF + MOG35-55" (days 14-52), MOG35-55 (days 16-52), and saline (days 13-52).

As previously shown for rat GMCSF-NAg TTV, the murine GMCSF-MOG TTV required physical linkage of GM-CSF and NAg domains for tolerogenic activity (Table 2 and Figure 4). Mice pretreated with GMCSF-MOG were largely protected from subsequent EAE. Only 3 of 16 mice pretreated with the TTV showed EAE, and the course was mild (Table 2 experiments 1 and 2 combined). Maximal scores for these mice were 2.0, 2.0, and 0.5. In contrast, mice pretreated with a mixture of GM-CSF and NAg had severe paralytic EAE. In this group, 15 of 16 mice had a maximal score of 4.0 whereas one mouse had a maximal score of 2.0. Mice pre-treated with GM-CSF did not differ in severity or time-course from saline-treated mice (Figure 4A, B) (note that the GM-CSF treatment group was not performed in experiment 2; Figure 4C, D). In this group, all mice had maximal scores of 3.5 (2 mice) or 4.0 (6 mice). Mice pre-treated with MOG35-55 had a slightly delayed onset but otherwise exhibited a full paralytic course of EAE (14 mice had maximal scores of ≥ 3.5 whereas 2 mice had scores of 0.5 and 2.0). Mice pre-treated with saline had severe EAE (14 of 16 had scores ranging from 3.0 to 5.0 whereas the two additional mice had scores of 2.0). Unlike the other pre-treatment groups, GMCSF-MOG prevented the EAE-associated loss of body weight (Figure 4B, D), and the covalent linkage of GMCSF with MOG35-55 was required for maintenance of normal body weight.

**Table 2:** GMCSF-MOG required physically-linked cytokine and NAg domains for tolerance induction

Exp. #	Treatment^a^	Mean cum. score	Median cum. score	Mean max. score	Median max. score	% mean initial weight	Incidence of EAE^b^	Mean # days with severe EAE^b^
1	Saline	71.9 ± 24.2	63.3	4.0 ± 0.7	4.0	82.1%	8 of 8	13.8 ± 10.0

1	GM-CSF	68.3 ± 23.6	64.0	3.9 ± 0.2	4.0	82.0%	8 of 8	13.6 ± 10.9

1	MOG35-55	102.1 ± 13.3	103.0	4.0 ± 0.0	4.0	81.8%	8 of 8	26.3 ± 3.8

1	GM-CSF + MOG	93.3 ± 19.5	90.5	4.3 ± 0.5	4.0	78.7%	8 of 8	23.1 ± 5.9

1	GMCSF-MOG	4.8 ± 13.0	0.0	0.3 ± 0.7	0.0	100.7%	2 of 8	0.0 ± 0.0

1	TTV vs Saline	p = 0.001		p < 0.001		p < 0.001	p = 0.007	p = 0.005

1	TTV vs GM-CSF	p = 0.004		p < 0.001		p < 0.001	p = 0.007	p = 0.030

1	TTV vs MOG35-55	p < 0.001		p < 0.001		p < 0.001	p = 0.007	p < 0.001

1	TTV vs "mixture"	p < 0.001		p < 0.001		p < 0.001	p = 0.007	p = 0.0002

2	Saline	74.1 ± 38.1	66.8	3.3 ± 0.9	3.5	89.5%	8 of 8	6.5 ± 8.8

2	MOG35-55	70.9 ± 44.5	81.5	3.2 ± 13	3.8	91.6%	8 of 8	7.6 ± 10.1

2	GM-CSF + MOG	78.4 ± 36.3	93.5	3.8 ± 0.7	4.0	84.9%	8 of 8	7.7 ± 6.3

2	GMCSF-MOG	3.0 ± 8.5	0.0	0.3 ± 0.7	0.0	100.1%	1 of 8	0.0 ± 0.0

2	TTV vs Saline	p = 0.001		p < 0.001		p = 0.005	p = 0.0014	p = 0.033

2	TTV vs MOG35-55	p = 0.001		p < 0.001		p = 0.030	p = 0.0014	p = 0.017

2	TTV vs "mixture"	p < 0.001		p < 0.001		p < 0.001	p = 0.0014	p = 0.004

^a ^Mice were treated with GMCSF-MOG, a mixture of murine GM-CSF and MOG35-55, the MOG35-55 peptide, GM-CSF, or saline subcutaneously on days -21, -14, and -7 (n = 8, all groups, 2 nanomoles per dose). Mice were then immunized on day 0 with 200 ug MOG35-55 in CFA plus Pertussis toxin (200 ng i.p.) on days 0 and 2. Table 2 and Figure 4 represent the same experiments. Data were analyzed as described for Table 1.

^b ^Severe EAE was defined as hindlimb paresis or paralysis (clinical score of 3.0 or greater).

**Figure 5:**
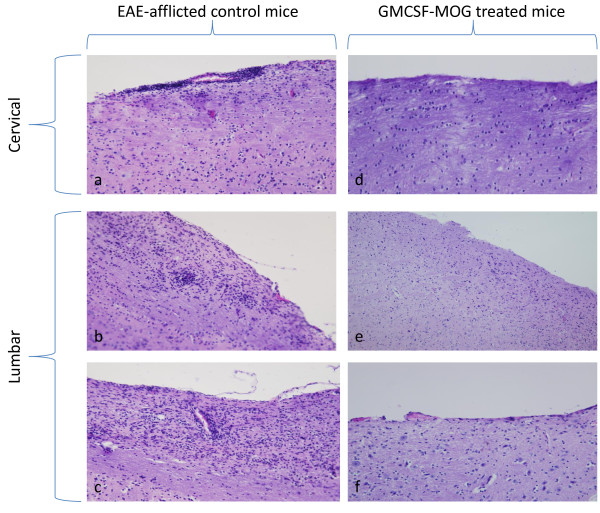
**GMCSF-MOG TTV prevented histological signs of EAE**. Shown are representative histological sections from mice from Figure 4, experiment 2. Mice from control groups that were afflicted with severe EAE had perivascular infiltrations of mononuclear cells typical of EAE in the cervical (a) and lumbar (b, c) regions of the spinal cord. In contrast, GMCSF-MOG treated mice did not show inflammatory lesions in the CNS, as portrayed by the cervical (d) and lumbar sections (e, f) of the spinal cord.

Mice successfully treated with GMCSF-MOG that had a clinical score of 0 did not exhibit histological evidence of EAE whereas EAE-afflicted control mice had abundant focal lesions of the CNS. These CNS lesions were marked by perivascular infiltration of mononuclear cells in white matter of the spinal cord (Figure 5). Overall, pretreatment with the GMCSF-MOG reduced EAE incidence, the cumulative score, the maximal disease score, weight loss, and the mean number of days that mice were afflicted by severe EAE. Due to the requirement for linked cytokine and NAg domains, GMCSF-MOG appeared to target the covalently-tethered NAg to APC in vivo as part of the tolerogenic mechanism. Overall, these data indicate that GMCSF-NAg TTV mediate antigen-targeting in both mice (Table 2) and rats [15].

### GMCSF-PLP pre-treatment prevented a subsequent phase of EAE

**Figure 6:**
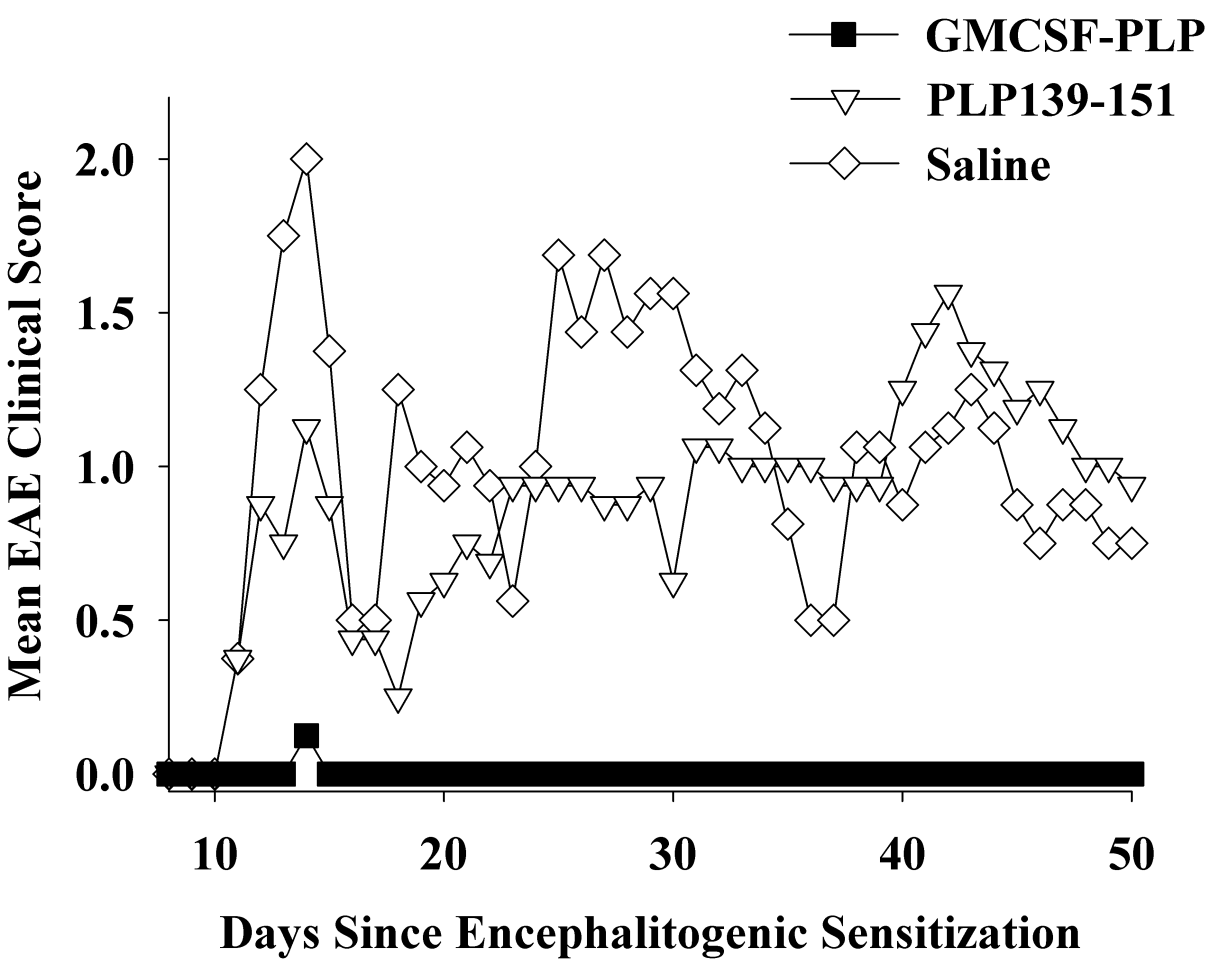
**The murine GMCSF-PLP TTV induced tolerance and prevented a subsequent episode of EAE**. SJL mice were treated with 2 nmoles of GMCSF-PLP (n = 8 each group), 2 nmoles of PLP139-151, or saline on days -21, -14, and -7 before active challenge on day 0 (200 ug of PLP139-151 in CFA). Table 3 and Figure 6 represent the same experiment. Daily clinical scores for mice treated with GMCSF-PLP(139-151) were significantly different from those treated with saline (days 12-15, 18-22, 25-34).

Given that GMCSF-NAg TTV were able to inhibit both monophasic (Lewis rats) and chronic progressive (C57BL/6 mice) models of EAE, an important question was whether GMCSF-NAg could inhibit relapsing-remitting EAE. Thus, GMCSF-PLP TTV was tested for tolerogenic activity in SJL mice (Table 3 and Figure 6). Mice were administered 2 nmoles GMCSF-PLP, 2 nmoles PLP139-151, or saline on days -21, -14, and -7 and then were challenged with 200 ug PLP139-151 in Complete Freund's Adjuvant (CFA) on day 0. One TTV-treated mouse exhibited EAE (incidence of 1/8, score of 1.0 for a total of 1 day) whereas mice pre-treated with either saline or PLP139-151 exhibited protracted, relapsing-remitting EAE. These data provide evidence that GMCSF-NAg TTV were effective in a second murine model of EAE.

**Table 3:** GMCSF-PLP prevented a subsequent bout of EAE induced by challenge with PLP139-151 in CFA

Pre-treatment^a^	Mean cum. score^b^	Median cum. score^b^	Mean max. score^b^	Median max. score^b^	% mean initial weight^b^	Incidence of EAE^c^	Mean # days with severe EAE^b^
Saline	43.1 ± 36.3	37.3	2.9 ± 1.5	3.5	87.1%	7 of 8	13.6 ± 12.8

PLP139-151	37.2 ± 35.4	31.0	2.5 ± 1.8	3.0	88.8%	6 of 8	12.4 ± 12.6

GMCSF-PLP	0.1 ± 0.4	0.0	0.1 ± 0.4	0.0	97.9%	1 of 8	0.0 ± 0.0

TTV vs saline	p = 0.002		p = 0.002		p = 0.004	p = 0.010	p = 0.047

TTV vs PLP	p = 0.011		p = 0.007		p = 0.016	p = 0.041	ns

^a ^SJL mice were treated with 2 nmoles of GMCSF-PLP (n = 8 each group), 2 nmoles of PLP139-151, or saline. Injections were subcutaneous in saline and were given on days -21, -14, and -7 (total combined dose of 6 nmoles) before active challenge on day 0 (200 ug of PLP139-151 in CFA). Table 3 and Figure 6 represent the same experiment. Data were analyzed as described for Table 1.

^b ^Mice were scored daily for clinical signs of EAE through day 50.

^c ^Incidence of EAE was the same as the incidence of severe EAE. Severe EAE was defined as ataxia through full hindlimb paresis or paralysis (clinical score of 2.0 or greater).

**Figure 7:**
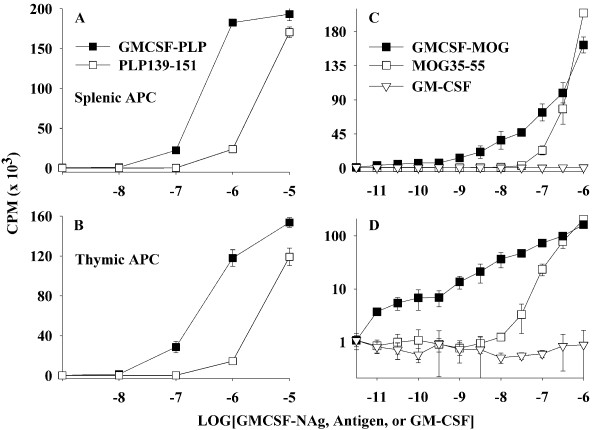
**The murine GMCSF-PLP and GMCSF-MOG TTV targeted NAg to APC for enhanced antigen presentation**. A PLP139-151-specific T cell line (A-B) or MOG35-55-specific T cell line (C-D) was cultured with irradiated SJL splenocytes (A), irradiated SJL thymocytes (B), or irradiated C57BL/6 splenocytes (C-D) as a source of APC together with designated concentrations (x-axis) of GMCSF-NAg, antigen, GM-CSF, or PLP139-151. C and D show the same data plotted on linear and logarithmic y-axes. Cultures were pulsed with [^3^H]thymidine during the last 24 hrs of a 72 hr culture. These data are representative of three experiments.

GMCSF-PLP was also a more potent as an antigen than the synthetic PLP139-151 peptide (Figure 7). The potency enhancement attributed to the covalent attachment with GM-CSF was approximately 10-fold and was independent of whether APC were obtained from the spleen (Figure 7A) or thymus (Figure 7B). GMCSF-MOG was also more potent than MOG35-55 for stimulation of a murine MOG-specific T cell line whereas GM-CSF did not stimulate proliferation (Figure 7C). The potency enhancement was evident over a wide concentration range and extended into the low picomolar range as was evident when the data were re-plotted on a logarithmic y-axis (Figure 7D). As noted previously (Figure 2), the same murine GMCSF-MOG TTV showed an approximate 1000-fold potency enhancement when tested in a rat T cell system.

### GMCSF-MOG was a therapeutic that inhibited the effector phase of an encephalitogenic attack

**Figure 8:**
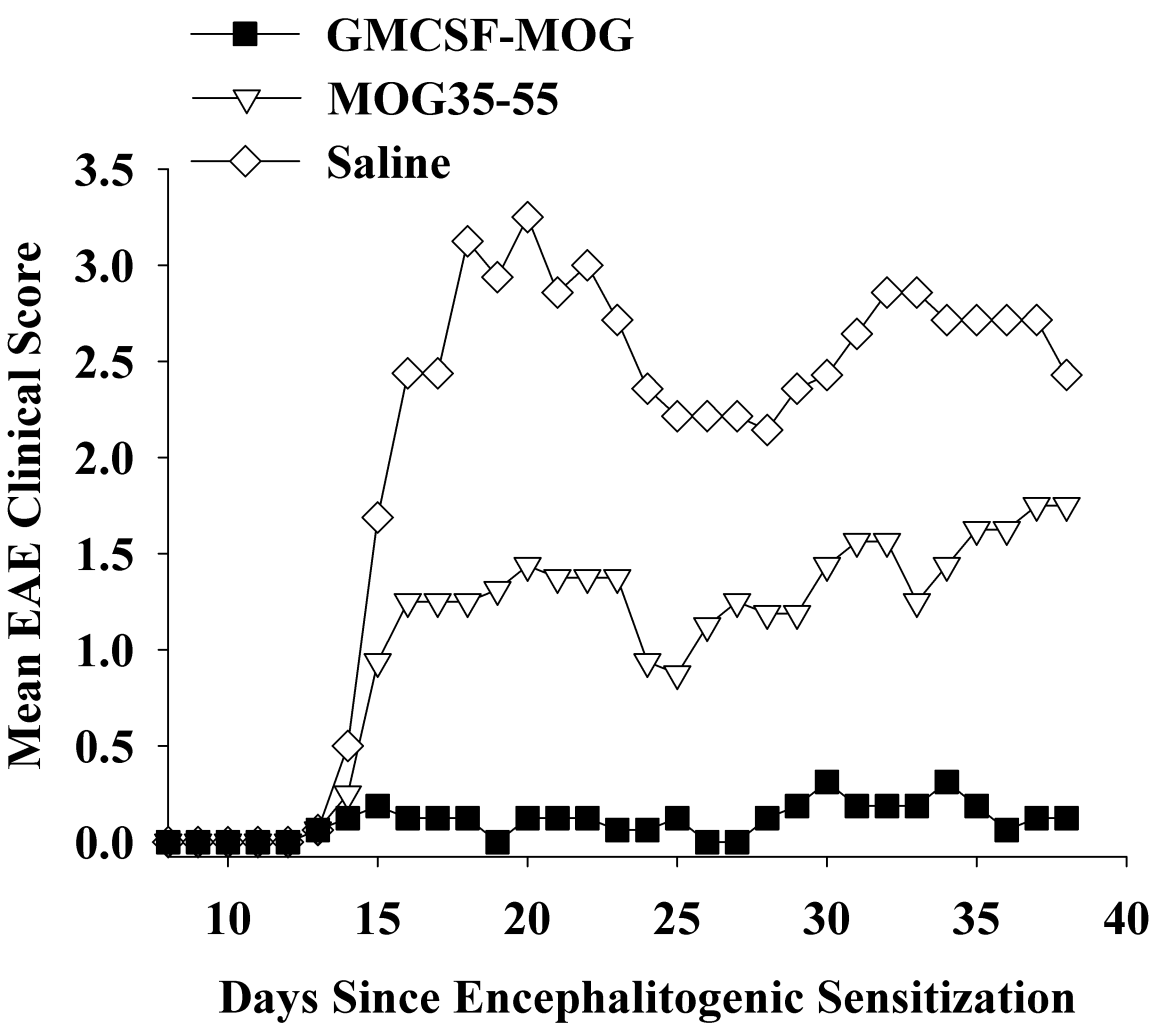
**GMCSF-MOG was an effective intervention that prevented progression of EAE**. Mice were immunized on day 0 with 200 ug MOG35-55 in CFA plus Pertussis toxin (200 ng i.p.) on days 0 and 2. Treatment was initiated when the first mice began showing clinical signs. Mice were matched for clinical signs and were injected subcutaneously with saline or 2 nanomoles of the synthetic peptide MOG35-55 or 2 nanomoles of the GMCSF-MOG TTV (in saline) on days 13, 15, 17, and 20. Table 4 and Figure 8 represent the same experiment. Daily clinical scores for mice treated with GMCSF-MOG significantly differed from those treated with MOG35-55 (days 16-38) or saline (days 15-38). Daily clinical scores for mice treated with MOG35-55 were significantly different from those treated with saline (days 18-22, 24, 32-36).

A central question was whether the GMCSF-MOG TTV can be used as an intervention in chronic EAE (Table 4 and Figure 8). Treatment was started on day 13 when the initial clinical signs of EAE first appeared in mice. The incidence of EAE was 1 of 8 mice for each group on day 13 (score was 1.0 for each mouse). Additional treatments were administered on days 15, 17, and 20. Administration of GMCSF-MOG halted development of EAE in a majority of mice whereas mice treated with MOG35-55 progressed to severe EAE. Of the GMCSF-MOG TTV-treated mice, 2 of 8 exhibited EAE, and one had clinical signs before initiation of treatment. Only 1 of 8 mice had a brief episode of severe EAE (score of 2 for a total of 2 days). The main conclusion was that the GMCSF-MOG TTV effectively stopped the progression of EAE. The rank order for inhibition of EAE was: GMCSF-MOG > MOG35-55 > saline. Compared to the saline-treated group, mice treated with MOG35-55 also had less severe EAE, although GMCSF-MOG had superior inhibitory efficacy compared to MOG35-55. These data indicate that GMCSF-MOG, and to a lesser extent, MOG35-55, inhibited the effector phase of EAE.

**Table 4:** The GMCSF-MOG TTV was a therapeutic treatment that blocked progression of actively-induced EAE

Treatment^a^	Mean cum. score	Median cum. score	Mean maximal score	Median maximal score	% mean initial weight	Incidence of EAE^b^	Mean # days with severe EAE^b^
Saline	62.4 ± 17.3	69.0	3.6 ± 0.4	3.5	77.6%	8 of 8	18.6 ± 7.3

MOG35-55	32.4 ± 24.7	26.5	2.3 ± 1.3	2.5	88.9%	7 of 8	9.1 ± 9.0

GMCSF-MOG	3.4 ± 6.3	0.0	0.4 ± 0.7	0.0	88.9%	2 of 8	0.3 ± 0.7

TTV vs Saline	p < 0.001		p < 0.001		p = 0.004	p = 0.007	p < 0.001

TTV vs MOG35-55	p = 0.001		p = 0.004		ns	p = 0.041	p = 0.007

MOG35-55 vs saline	p = 0.024		p = 0.010		p = 0.004	ns	p = 0.036

^a ^Mice were immunized on day 0 with 200 ug MOG35-55 in CFA plus Pertussis toxin (200 ng i.p.) on days 0 and 2. Treatment was initiated when the first mice began showing clinical signs. Mice were matched for clinical signs and were injected subcutaneously with saline or 2 nanomoles of the synthetic peptide MOG35-55 or 2 nanomoles of the GMCSF-MOG TTV (in saline) on days 13, 15, 17, and 20. Mice were scored daily for clinical signs of EAE through day 38. Table 4 and Figure 8 represent the same experiment. Data were analyzed as described for Table 1.

^b ^Severe EAE was defined as a clinical score of 2.0 or greater.

**Figure 9:**
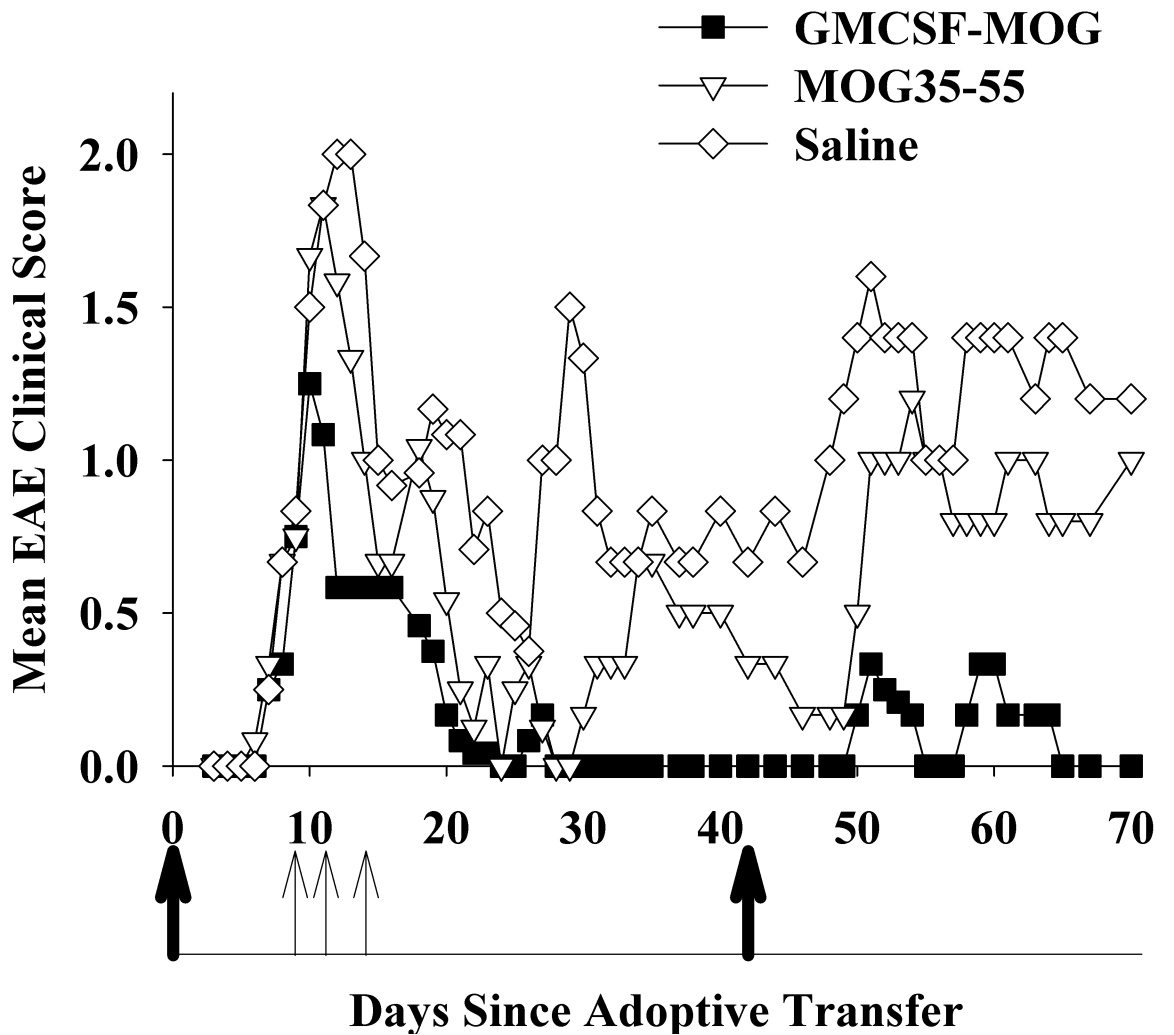
**GMCSF-MOG promoted tolerogenic activity despite ongoing inflammation in the CNS**. Passive EAE was induced in C57BL/6 mice by adoptive transfer of activated MOG35-55-specific T cells on day 0 and by injection of Pertussis toxin on days 0 and 2. Mice were matched for EAE severity on day 9 (mean maximal severity of 0.8 and an incidence of 100% for all groups), and were then treated with GMCSF-MOG TTV or controls (subcutaneous in saline) on day 9 (4 nanomoles), day 11 (4 nanomoles), and day 14 (2 nanomoles). Mice were challenged with MOG35-55 in CFA on day 42 and Pertussis toxin was injected i.p. on days 42 and 44 to elicit a second bout of active EAE. Table 5 and Figure 9 represent the same experiment. Daily clinical scores for mice treated with GMCSF-MOG were significantly different from those treated with "MOG35-55" (days 54-57, 61-63, 65-70) or saline (days 28-58, 61-70). Thick arrows represent day 0 (adoptive transfer) and day 42 (active immunization). Thin arrows represent days that mice were treated with GMCSF-MOG, MOG35-55, or saline.

GMCSF-MOG was also tested as a therapeutic agent in an alternative model (Table 5 and Figure 9). EAE was passively-induced by adoptive transfer of encephalitogenic T cells, and then, after onset of clinical signs, three treatments were administered on days 9, 11, and 14. Because all mice had EAE before the initiation of treatment, the incidence of EAE in all groups was 100%. Following partial recovery from the initial passive bout of EAE, mice were actively challenged with MOG35-55 in CFA on day 42 but were not given any additional treatment with TTV. The GMCSF-MOG TTV, when administered on days 9, 11, and 14, blunted the initial bout of passive EAE, inhibited residual disease, and attenuated the subsequent bout of active EAE. Overall, these data show that therapeutic administration of TTV during the initial passively-induced bout of EAE had a pronounced inhibitory effect on the subsequent active induction of EAE as measured by both cumulative and maximal disease scores. These data indicate that tolerogenic activity can be initiated in peripheral lymphoid tissues despite ongoing inflammation in the CNS.

**Table 5:** The GMCSF-MOG TTV was an intervention that reversed an established course of chronic EAE

Treatment^a^	Mean cumulative score^b^	Median cumulative score^b ^	Mean maximal score^b^	Median maximal score^b ^	Incidence of EAE	Mean # days with severe EAE
Saline	47.9 ± 19.8	41.8	2.7 ± 0.5	3.0	6 of 6	20.0 ± 1.4

MOG35-55	27.6 ± 17.9	20.0	1.8 ± 0.8	2.0	6 of 6	13.8 ± 6.5

GMCSF-MOG	6.8 ± 4.6	4.5	0.8 ± 0.6	0.5	6 of 6	1.7 ± 2.7

TTV vs MOG35-55	p = 0.008		p = 0.034		----	p = 0.051

TTV vs saline	p < 0.001		p < 0.001		----	p = 0.001

^a ^Passive EAE was induced in C57BL/6 mice by adoptive transfer of activated MOG35-55-specific T cells on day 0 and by injection of Pertussis toxin on days 0 and 2. Mice were matched for EAE severity on day 9 (mean maximal severity of 0.8 and an incidence of 100% for all groups), and were then treated with GMCSF-MOG TTV or controls (subcutaneous in saline) on day 9 (4 nanomoles), day 11 (4 nanomoles), and day 14 (2 nanomoles). Mice were challenged with MOG35-55 in CFA on day 42 and Pertussis toxin was injected i.p. on days 42 and 44 to elicit a second bout of active EAE. Table 5 and Figure 9 represent the same data.

^b ^The cumulative and maximal scores were assessed from days 12-70, and the incidence of severe EAE was assessed from days 15-70. An alternative clinical scale was used for this experiment (0.5, tail involvement; 1.0, ataxia, 2.0 partial paralysis, 3.0, full paralysis), and mean number of days with severe EAE was assessed from days 44-70 during the active challenge. Severe EAE was defined as a clinical score of 1.0 or greater.

## Discussion

### GM-CSF as a tolerogenic fusion partner

The purpose of this study was to assess whether a murine TTV comprised of mouse GM-CSF and encephalitogenic epitopes from MOG or PLP effectively controlled chronic forms of EAE in mice. A previous study showed that a single-chain TTV comprised of rat GM-CSF and the major encephalitogenic epitope of MBP was effective for controlling the monophasic form of EAE in the Lewis rat [15]. Chronic and monophasic models of EAE however differ substantially in mechanism. Particularly, monophasic forms of EAE may be self-limiting due to robust regulatory responses that enable recovery and prevent relapses. In contrast, chronic forms of EAE appear to feature inefficient regulatory mechanisms that cannot reverse CNS inflammation. To assess this question, we derived murine GMCSF-MOG(35-55) and GMCSF-PLP(139-151) TTV and tested these fusion proteins in the C57BL/6 chronic progressive model and the SJL relapsing-remitting model of EAE, respectively. When administered as three injections before active immunization, GMCSF-MOG prevented chronic progressive EAE in C57BL/6 mice (Tables 1, 2, Figures 3, 4), and GMCSF-PLP prevented relapsing-remitting EAE in SJL mice (Table 3 Figure 6), respectively. Overall, these data indicate that specific GMCSF-NAg TTV were effective in three models of EAE, including both mouse and rat species as well as monophasic, chronic progressive, and relapsing remitting models of EAE.

Rat GMCSF-NAg TTV (Figure 2A), the murine GMCSF-PLP (Figure 7A, B), and GMCSF-MOG (Figure 7C, D) exhibited enhanced antigen presentation compared to the respective NAg. Due to the full cross-species reactivity of GM-CSF, murine GMCSF-MOG was targeted to both rat (Figure 2B) and murine APC (Figure 7C, D) to mediate enhanced antigen presentation to rat and mouse MOG-specific T cells. These data reveal that the NAg domain is efficiently targeted, processed, and presented to T cell clones specific for the respective NAg. An important question was whether covalent linkage of the GM-CSF and NAg domains that was required for antigen targeting and enhanced presentation of NAg by DC was also required for tolerogenic activity in vivo. In Lewis rats, physical linkage of the GM-CSF and NAg domains was needed for prevention and therapy of EAE [15]. To assess this question for murine TTV, we focused on the C57BL/6 model of EAE. As shown in Table 2 and Figure 4, tolerogenic activity of GMCSF-MOG was entirely contingent upon the covalent linkage of GM-CSF and MOG35-55 because administration of the two agents as an equimolar mixture of separate molecules did not suppress disease. Pre-treatment with either GM-CSF alone or NAg alone also did not affect EAE. The requirement for physical linkage of the GM-CSF and NAg domains for tolerogenic activity in vivo provided evidence that a mechanism of antigen targeting was required for inhibition of EAE.

GMCSF-MOG also halted progression of EAE when administration of the TTV was initiated at disease onset (Table 4 Figure 8). Treatment was initiated when the mice first began to show clinical signs on day 13. Hence, treatment was initiated during early onset at a time when effector T cells were first transitioning to the CNS. Additional treatments were given on days 15, 17, and 20. Administration of GMCSF-MOG at these time-points blocked the progression of disease. In contrast, mice treated with MOG35-55 or saline progressed to paralytic disease, although mice treated with MOG35-55 had a less severe course than those treated with saline. These data indicated that GMCSF-MOG inhibited established effector mechanisms underlying progression of EAE. GMCSF-MOG was also able to attenuate a model of passive EAE that was subsequently boosted by an active challenge (Table 5 Figure 9). In this case, treatment with GMCSF-MOG was initiated when most mice were already afflicted with EAE. GMCSF-MOG accelerated recovery and blunted the subsequent active induction of EAE. Thus, even during peak CNS inflammation in a prevailing immunogenic environment, GMCSF-MOG attenuated disease. Overall, these data indicated that GMCSF-MOG is tolerogenic in both quiescent, non-inflammatory environments as well as activated, pro-inflammatory environments.

### Targeting of NAg to DC is a potent means of tolerance induction

Recombinant antibody-antigen fusion proteins specific for DEC-205 (CD205) also targeted covalently tethered foreign peptide antigen to DC for enhanced presentation by a mechanism that resulted in antigen-specific tolerance [19,20]. When antigen was targeted to DC, transgenic T cells initially exhibited a burst of antigen-specific proliferation, but the response collapsed and tolerance emerged. Similar antibody-antigen fusion proteins were also used to prevent the induction of EAE [21,22]. A recombinant anti-DEC205 antibody was expressed that contained the MOG35-55 peptide as the C-terminus. When administered 7 days before encephalitogenic challenge, this anti-DEC205-MOG fusion protein blocked the subsequent development of EAE. The anti-DEC205-MOG fusion protein did not cause clonal deletion of MOG-specific T cells but rather elicited clonal anergy in association with elevated CD5 expression. Likewise, a fusion protein comprised of the anti-DEC-205 mAb and the PLP139-151 epitope inhibited the subsequent induction of EAE in SJL mice. Tolerance was associated with deletion and anergy in pathogenic CD4^+ ^T cell subsets along with expansion of regulatory T cell subsets. Overall, these studies provided evidence that targeting of encephalitogenic NAg to DC by use of anti-DEC205-NAg fusion proteins can lessen susceptibility to EAE. These studies lend credence to the hypothesis that efficient presentation of self antigens by DC may be conducive for restoration of self tolerance as a means to inhibit autoimmune demyelination.

### Advantages of GM-CSF as a tolerogenic fusion partner for NAg

GM-CSF is well-known as a pivotal cytokine that drives differentiation of DC, including tolerogenic or regulatory DC subsets. These APC subsets are important for induction and maintenance of self tolerance [23-34]. GM-CSF directly promotes differentiation of DC subsets that in turn facilitate Treg differentiation and antigen-specific tolerance. Likewise, GM-CSF promotes differentiation of myeloid derived suppressor cells that secrete anti-inflammatory cytokines, mediators such as nitric oxide, and enzymes such as IDO to downregulate T cell responses and confer tolerance [35-42]. Due to the emerging recognition that GM-CSF and DC have important regulatory dimensions, substantial emphasis for development of GM-CSF-based cancer vaccines is focused on breaching Treg-based regulatory networks [43,44].

GM-CSF is now recognized as a potent regulatory cytokine able to ameliorate disease in several mouse models of autoimmunity. GM-CSF, when delivered alone without a corresponding antigen, inhibited disease in experimental autoimmune thyroiditis (EAT) [45-49], experimental autoimmune myasthenia gravis (EAMG) [50-52], and type I autoimmune diabetes (T1D) [53-55]. In CBA/J mice, treatment with GM-CSF before or after immunization with thyroglobulin attenuated the severity of EAT and reduced thyroglobulin-specific T cell autoimmunity. Inhibition of EAT was contingent upon enhanced IL-10 production and increased activities of tolerogenic myeloid DC and CD4^+^CD25^+ ^Foxp3^+ ^regulatory T cells. Likewise, GM-CSF treatment was effective for both prevention and suppression of EAMG by a mechanism associated with the reduction of autoreactive T cell responses, lowered serum autoantibody production, enhanced production of IL-10, enhanced suppressive activity of myeloid DC, and expansion of Foxp3^+ ^Treg cells. GM-CSF treatment of NOD mice also inhibited spontaneous development of autoimmune diabetes by induction of regulatory CD11c^+ ^DC and Foxp3^+ ^T cells. In accordance with the observation that GM-CSF treatment inhibited T1D, a genetic deficiency of GM-CSF enabled disease [56,57]. That is, T1D was evident in aged C57BL/6 mice deficient in GM-CSF, and to a greater extent, in aged mice deficient in both GM-CSF and IL-3. These double-deficient mice exhibited a SLE-like disease together with insulitis, loss of insulin-producing beta cells, and dysregulated blood glucose levels. GM-CSF-deficient myeloid cells exhibited an impaired phagocytosis of apoptotic cells [57,58]. Suboptimal phagocytosis of apoptotic bodies may restrict an important source of self peptides needed for diversification of the Treg repertoire. Thus, defects in GM-CSF-mediated phagocytosis and clearance of apoptotic cells may be associated with insufficient development of the Treg repertoire and development of autoimmune inflammatory disease. GM-CSF treatment appears to reverse these defects to restore self tolerance. Overall, these studies indicate that GM-CSF is pivotal for differentiation of tolerogenic DC that in turn promote regulatory T cell activities necessary for immune homeostasis.

The common link of these studies with our study of GMCSF-NAg is that GM-CSF, either as an independent regulatory cytokine or as a tolerogenic fusion partner, had the essential activities that restored tolerance to relevant self-antigens and thereby inhibited autoimmune disease. The requirement for coupling of GM-CSF to the relevant self-antigen in EAE (as opposed to EAT, EAMG, and T1D) may reflect differences in underlying pathogenic mechanisms, drug treatment regimens, and/or bioavailability of the relevant antigens. One of the main advantages of coupling self antigens to GMCSF is that GMCSF would predictably target that antigen with high efficiency to the regulatory APC subsets most directly implicated in self tolerance, potentially including a continuum of tolerogenic DC, myeloid derived suppressor cells, and immature or semi-mature DC. Targeting self-antigen to these APC may represent a key innovation toward achieving high-efficiency presentation of self antigens by APC known to enhance Treg responses, reverse autoimmune disease, and restore self-tolerance.

## Conclusions

The GM-CSF domain of GMCSF-NAg TTV serves as a 'gateway' to the immune system by catalyzing high-efficiency uptake of the covalently-tethered NAg for presentation by myeloid APC. Overall, this study supports the possibility that GM-CSF fusions with 'self' myelin epitopes may represent a generalized approach for the induction of tolerance to CNS myelin antigens.

## Methods

### Structure and purification of recombinant proteins

Expression systems for three murine GM-CSF based fusion proteins (GM-CSF, GMCSF-MOG, and GMCSF-PLP) were derived for this study. Murine GM-CSF contained the native signal sequence "M-A-W-L-Q-N-L-L-F-L-G-I-V-V-Y-S-L-S" with a non-native alanine at the second position to accommodate a Kozak translation initiation site. The C-terminus had an 8-histidine affinity tag without an intervening linker. The GMCSF-MOG protein was identical except that the MOG35-55 "M-E-V-G-W-Y-R-S-P-F-S-R-V-V-H-L-Y-R-N-G-K" was encoded between the native GM-CSF and the 8-histidine C-terminus. The GMCSF-PLP protein contained the PLP139-151 sequence "H-S-L-G-K-W-L-G-H-P-D-K-F" rather than MOG35-55 and was otherwise identical. PCR products were assembled by overlap extension PCR and were inserted into the pCEP4 expression vector (Invitrogen) for transient expression in human embryonic kidney (HEK) 293 cells. Expression supernatants containing the recombinant proteins were concentrated by ultrafiltration and were purified by affinity chromatography based upon binding to a single chain (scFv) anti-6 his antibody immobilized on a chitin resin [59,60]. After elution, the fusion proteins were subjected to a final affinity chromatography step on Ni-NTA agarose columns (Qiagen, Valencia, CA, USA). Proteins were concentrated and diafiltrated in Amicon Ultra-15 centrifugal filter devices. Protein quantity was assessed by absorbance at 280 nm. Purity was assessed by SDS-PAGE. GMCSF-GP(69-87), referred to as GMCSF-NAg in [15], was a fusion of rat GM-CSF with the 69-87 encephalitogenic sequence of guinea pig (GP) myelin basic protein "Y-G-S-L-P-Q-K-S-Q-R-S-Q-D-E-N-P-V-V-H". The sequence of the GP69-88 peptide was: Y-G-S-L-P-Q-K-S-Q-R-S-Q-D-E-N-P-V-V-H-F. The C-terminal Phe residue is not part of the major encephalitogenic epitope recognized by Lewis rat T cells [61,62].

### Animals and reagents

C57BL/6 and SJL mice were housed at East Carolina University Brody School of Medicine. Animal care and use was performed in accordance with approved animal use protocols and guidelines of the East Carolina University Institutional Animal Care and Use Committee. Synthetic MOG35-55 and PLP139-151 peptides were obtained from University of North Carolina Microprotein Sequencing & Peptide Synthesis Facility (Chapel Hill, NC).

### Cell lines and culture conditions

Cell lines were cultured in complete RPMI medium [10% heat-inactivated fetal bovine serum, 2 mM glutamine, 100 ug/ml streptomycin, 100 U/ml penicillin (Whittaker Bioproducts, Walkersville, MD), 50 uM 2-ME (Fisher Scientific, Pittsburgh, PA)]. The rat MOG(35-55)-specific T cell line was a primary, IL-2 dependent line derived from Lewis rats sensitized with MOG35-55 in Complete Freund's Adjuvant (CFA). Murine T cell lines were derived from C57BL/6 and SJL mice sensitized with 200 ug of MOG35-55 or PLP139-151 in CFA, respectively. T cell lines were propagated in complete RPMI supplemented with recombinant rat IL-2 (0.4% v/v Sf9 supernatant) [63]. The 11B11 hybridoma (ATCC HB-188), FDC-P1 (ATCC CRL-12103), and CTLL (ATCC TIB-214) lines were obtained from the American Type Culture Collection. 11B11 was a hybridoma that secreted a rat anti-mouse IL-4 monoclonal antibody. FDC-P1 and CTLL lines were used as indicator cells for measurement of GM-CSF and IL-2. The FDC-P1 line was derived from DBA/2 bone marrow cells, and the CTLL line was an IL-2-dependent murine T cell line.

### Bioassays

To measure proliferation, cultures were pulsed with 1 uCi of [^3^H]thymidine (6.7 Ci/mmol, Perkin Elmer, Waltham, MA, USA) during the last 24 hr of a 72 hr culture. Cultures were harvested onto filters by use of a Tomtec Mach III harvester (Hamden, CT, USA). [^3^H]thymidine incorporation into DNA was measured by use of a Wallac 1450 Microbeta Plus liquid scintillation counter (Perkin Elmer, Waltham, MA). To measure IL-2 production, culture supernatants (100 ul) were transferred from the assay plate to a replicate plate, and CTLL cells (10,000/well) in complete RPMI were added to each well. Cultures were pulsed with MTS/PMS [3-(4,5-dimethylthiazol-2-yl)-5-(3-carboxymethoxyphenyl)-2-(4-sulfophenyl)-2H-tetrazolium, inner salt; MTS] and phenazine methosulfate (Promega, Madison, WI) during the last 24 hr of a 72 hr culture, followed by measurement of 490 nm absorbance. Error bars represented standard deviations of triplicate or quadruplet sets of wells. Bioassays portrayed in Figures 2 and 7 are representative of at least three experiments.

### Induction and treatment of EAE

CFA was prepared by mixing Incomplete Freund's Adjuvant with heat-killed *Mycobacterium tuberculosis *(4 mg/ml) (BD Biosciences, Franklin Lakes, NJ). The CFA adjuvant was mixed 1:1 with the designated dose of antigen in saline and emulsified by sonication. Active induction of EAE with synthetic peptides in CFA was performed by subcutaneous injection across the back. Each mouse received three separate injections (~ 0.033 ml per injection) for a total injection volume of 0.1 ml per mouse. Passive EAE was induced by adoptive transfer of activated encephalitogenic T cells. Donor mice were actively sensitized with MOG35-55 in CFA. After 10-14 days, draining lymph nodes and splenocytes were harvested from donor mice and were cultured with MOG35-55, IL-12, and the 11B11 anti-IL-4 monoclonal antibody. After 3 days of culture, activated T cells were injected into recipients (0.5 ml total volume by intraperitoneal injection). In designated protocols, sequential passive and active EAE was induced in the same mice. The first bout of EAE was induced by adoptive transfer of activated encephalitogenic T cells. After peak disease, the mice showed varying degrees of recovery. To elicit a second uniformly-intense second bout of EAE, mice were actively challenged with MOG35-55 in CFA. For passive and active induction of EAE, C57BL/6 mice received an injection of 200 ng of Pertussis toxin (List Biological Labs, Inc., Campbell, CA) in PBS i.p. on the day of immunization and again 48 hours later. Fusion proteins or controls were administered to mice anesthetized by isoflurane (Abbott Laboratories, Chicago, IL, USA). Fusion proteins, synthetic peptides, or combinations were solubilized in saline and injected subcutaneously in 0.1 - 0.2 ml volumes. Injection sites showed no signs of inflammation.

### Assessment of Clinical EAE

EAE was scored by the following scale: 0, no disease; 0.5, partial paralysis of tail without ataxia; 1.0, flaccid paralysis of tail or ataxia but not both; 2.0, flaccid paralysis of tail and ataxia or impaired righting reflex; 3.0, partial hind limb paralysis marked by inability to walk upright but with ambulatory rhythm in both legs; 3.5, same as above but with full paralysis of one leg; 4.0, full hindlimb paralysis; 5.0, total hindlimb paralysis with forelimb involvement or moribund. A score of 5.0 was a humane endpoint for euthanasia. In Table 5 an alternative scoring scale was used (see footnote) representing the previous scale routinely used in the lab. Disease measurements included incidence of EAE, mean and median cumulative score, mean and median maximal scores, percent weight loss, incidence of severe EAE, mean number of days with severe EAE, and clinical scores for each day. Cumulative scores were calculated by summing daily scores for each mouse. Maximal scores were calculated as the most severe EAE score for each mouse, including all mice within a group. Mice that had a score of zero (that did not exhibit EAE) were included in the calculation of the mean maximal score. Cumulative and maximal scores were analyzed by nonparametric ANOVA based on ranked scores. "Percent mean initial weight" and "number of days with severe EAE" was analyzed by parametric ANOVA. The threshold for severe EAE was based on the average maximal severity for each experiment and was defined in each table legend. ANOVA was assessed with a Bonferroni post hoc test. "Incidence of EAE and incidence of severe EAE" was analyzed pair-wise by Fisher's Exact Test.

### Histological assessment of EAE

After humane euthanasia, brain and spinal cord were fixed in 4% paraformaldehyde. Tissues were dehydrated in graded ethanol, cleared with citriSolv (Fisher Scientific, Pittsburgh, PA), and embedded in a 100% paraffin block. Parasagittal (10 micron thick) sections were mounted onto glass slides and stained with hematoxylin and eosin. Sections were imaged with a spot insight digital camera connected to an Olympus Bx51 microscope at 20× magnification and were scored for perivascular lesions of EAE.

## Abbreviations

APC: antigen presenting cells; CFA: Complete Freund's Adjuvant; DC: dendritic cells; EAE: experimental autoimmune encephalomyelitis; GMCSF-MOG: fusion protein comprised of GM-CSF and MOG35-55; GMCSF-PLP: fusion protein comprised of GM-CSF and PLP139-151; GM-CSF: granulocyte-macrophage colony stimulating factor; MBP: myelin basic protein; MOG: myelin oligodendrocyte glycoprotein; MS: multiple sclerosis; Nag: neuroantigen; PLP: proteolipid protein; TTV: tolerogenic therapeutic vaccine.

## Authors' contributions

DJA performed and provided oversight for expression, purification, and bioassay of recombinant proteins and conducted many of the EAE experiments. JLB also performed the expression, purification, and bioassay of recombinant proteins, and conducted several of the preliminary EAE experiments. DAM constructed the expression plasmids and pioneered expression and bioassay of the recombinant proteins. NT conducted the histological analysis of EAE. NT, SCR, and ADC performed bioassays that provided evidence of 'antigen-targeting'. All authors were involved in the interpretation of data, provided important intellectual insight, and contributed to the critical revision of the manuscript. MDM conceptualized, designed, and coordinated the project, drafted the manuscript, and contributed to data acquisition. All authors read and approved the final manuscript.
